# A non-interleaved bidirectional Janus metasurface with full-space scattering channels

**DOI:** 10.1515/nanoph-2022-0292

**Published:** 2022-08-05

**Authors:** Guanyu Shang, Guangwei Hu, Chunsheng Guan, Yue Wang, Kuang Zhang, Qun Wu, Jian Liu, Xue-Mei Ding, Shah Nawaz Burokur, Haoyu Li, Xumin Ding, Cheng-Wei Qiu

**Affiliations:** Advanced Microscopy and Instrumentation Research Center, Harbin Institute of Technology, Harbin 150080, China; Department of Electrical and Computer Engineering, National University of Singapore, Singapore 117583, Singapore; Department of Microwave Engineering, Harbin Institute of Technology, Harbin 150001, China; LEME, UPL, Univ Paris Nanterre, F92410 Ville d’Avray, France; State Key Laboratory of Robotics and Systems, Harbin Institute of Technology, Harbin 150080, China

**Keywords:** bidirectional, full space, metasurface, non-interleaved

## Abstract

Metasurfaces have attracted broad interest thanks to their unprecedented capacity for electromagnetic wavefront manipulation. The compact, ultrathin and multifunctional metasurface calls for novel design principles. Here, we propose and experimentally demonstrate a non-interleaved and non-segmented bidirectional Janus metasurface that encodes multiple functionalities in full-space scattering channels with different propagation directions and polarization in the microwave region. Specifically, by rotating and adjusting the elementary double-arrow-shaped structure within the same meta-atom, the independent phase control can be achieved in both cross-polarized transmission and co-polarized reflection components under oppositely directed incident waves. Our metasurface with broken mirror symmetry can fully exploit four independent information channels under opposite propagation directions. A series of proof-of-concept is constructed to validity of our methodology, and the simulations and experimental results further show that the proposed non-interleaved bidirectional metasurface can provide an attractive platform for various applications, ranging from structured light conversion, optical imaging, multifunctional optical information processing and others.

## 1 Introduction

Metasurface, composed of two-dimensional artificial meta-atoms, offers the complete control of light, including amplitude [1–3], phase [4–6] and polarization [7–10] within a platform of various merits such as thinness, easy fabrication and low loss, compared to conventional optical elements. A plethora of exciting applications are demonstrated, such as optical vortex beam generation [11–13], energy absorption [14, 15] beam deflection [16, 17] and all-optical image processing [18, 19], just to name a few. The demand to process the increased information loads with more compact devices in modern optics calls for multifunctional metasurfaces. For instance, the transmission-type metasurface can perform the multiplexing of wavelength [20–23], polarization [24–28], angle [29–31] and angular momentum [32–35] of light, thus enriching the information processing capabilities. Recently, the capacity limit of a single transmissive metasurface as characterized with the Jones matrix has been demonstrated [36], which leads to a natural question of whether one can further add more capacity atop it. To answer this, we note such claims only hold true under the assumption of a metasurface working in half-space, either in reflection or transmission sides, while leaving full-space scattering channels overlooked.

Extensive efforts have focused on designing a metasurface with both transmissive and reflective functionalities, which, for instance, can be achieved at different frequencies [37, 38]. More recently, the seemingly contradictory diffuse reflection and distortion-free transmission are demonstrated in a single random-flip metasurface exploiting the optical reciprocity and spatial inversion [39], showing new freedoms in full-space control of light. Other approaches can include the reconfigurable metasurfaces using, for instance, diodes to configure the full-space scattering channels [40–43]. Those results suggest the possibilities to break the capability limit of a single-channel metasurface characterized by the Jones matrix, which however remains unexplored.

**Figure 1:**
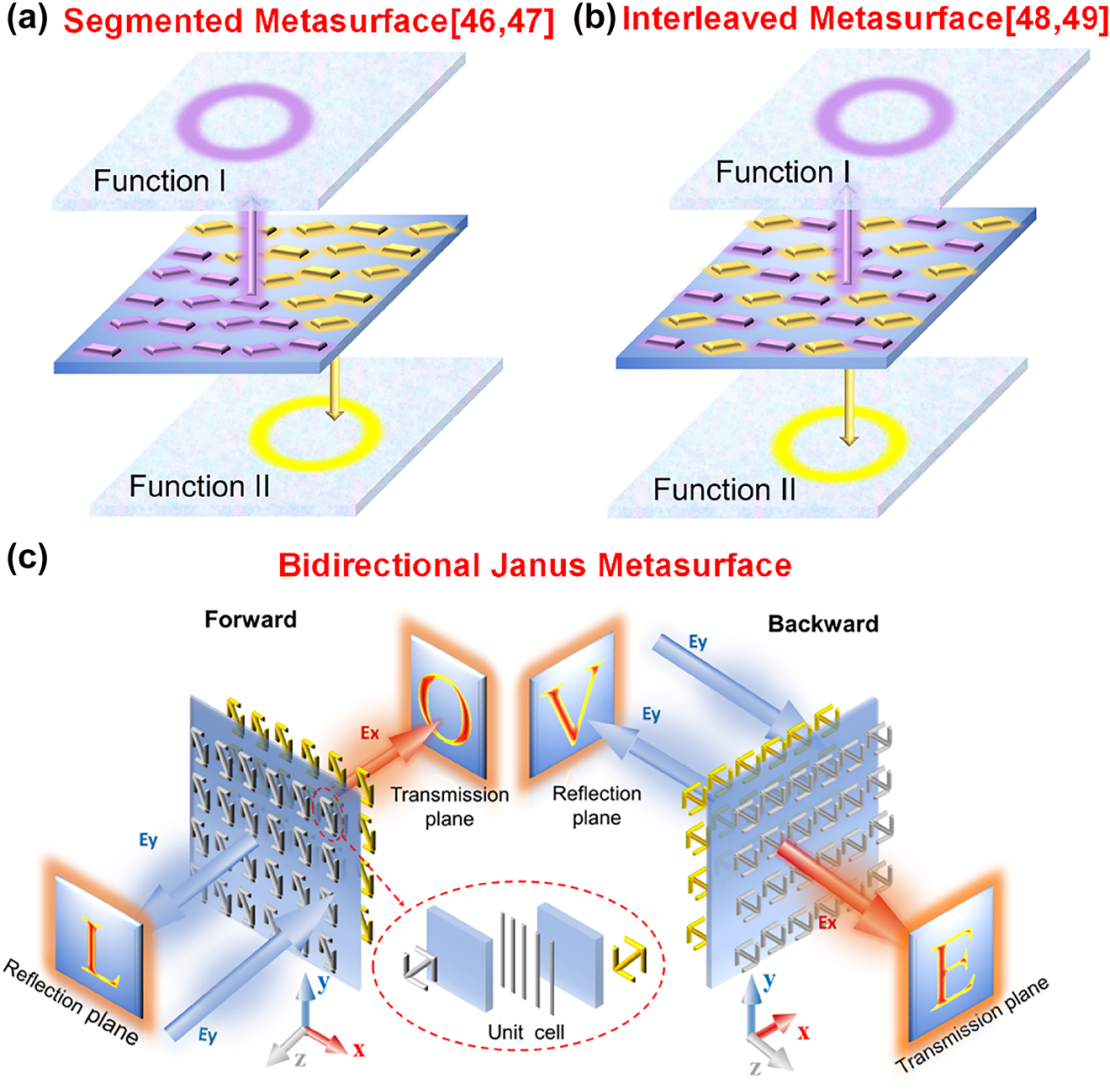
Schematic diagram of the multifunctional metasurface. (a) Illustration of the segmented metasurface. (b) Illustration of the interleaved metasurface. (c) Schematic illustration of the proposed non-interleaved multifunctional metasurface.

Even more degrees of freedom of metasurface design can come from the introduction of chiral and anisotropic meta-atoms, which induces asymmetric responses and distinguished functionalities when an incident from different sides. Such direction-selective planar metasurfaces, also known as Janus metasurfaces mimicking the two-faced Janus God, have been studied for a wide range of applications [44, 45]. Several strategies are proposed to leverage such effect. A single-layer all-dielectric metasurface composed of two segments, each one operating for incident waves from a specific side, can render distinguished spatial phase distributions [46]. Recently, we exploited the triangular segmented metasurface to complete the reflection and transmission channels, but the realization of its bidirectional function is actually equivalent to the splicing effect of two metasurfaces [47]. One triangular region plays a role under forward incidence, while the other provides the effect of reflection and transmission functions under the backward incident. In addition, the imaging surface is limited to the corresponding unit cell arrangement zone. Besides, a three-dimensional (3D) Janus metamaterial can show direction-controlled polarization multiplexing [48]. More recently, Chen et al. [49] adopted unidirectional propagating unit cells with stacked elements arranged in a chessboard-like distribution to achieve directional Janus metasurface with two asymmetry transmission channels. However, no matter the segmented (Figure 1a) or interleaved (Figure 1b) metasurface, only half of the meta-cells in the metasurface are used for each direction. Therefore, it is still a challenge to make full use of each unit cell on the metasurface to realize the control of multiple channels with opposite incident propagation directions at the same frequency.

Herein, we demonstrate a transmission-reflection-integrated Janus metasurface, supporting independent functions in both reflection and transmission channels with different incident directions at the same frequency. Importantly, meta-atoms with multilayer composites have broken mirror symmetry, which allows different transmission performances propagating in opposite directions for our demonstrated extreme wave manipulations. Besides, all meta-atoms contribute to the scattering, thus free of segmented or interleaved arrangement (Figure 1a and b) and suggesting the full utilization of the metasurface. As a proof of concept, for the forward and backward propagations of the *y*-polarized incidence, co-polarized reflection and cross-polarized transmission channels in two directions can be independent control and produce four different holographic imaging effects with broken mirror symmetry (Figure 1c). Moreover, protected by reciprocity, the reflected cross-polarized component can show the same holography with the transmitted cross-polarized component when propagation direction changes, suggesting six concurrent operation channels in our designs. Our Janus metasurface with the integration of multifunctional channels in full spaces suggests a new regime of meta-devices with high function capacity and compactness, of great potential in information encryption, optical anti-counterfeiting and others.

## 2 Bidirectional Janus metasurfaces with the non-interleaved assignment

**Figure 2:**
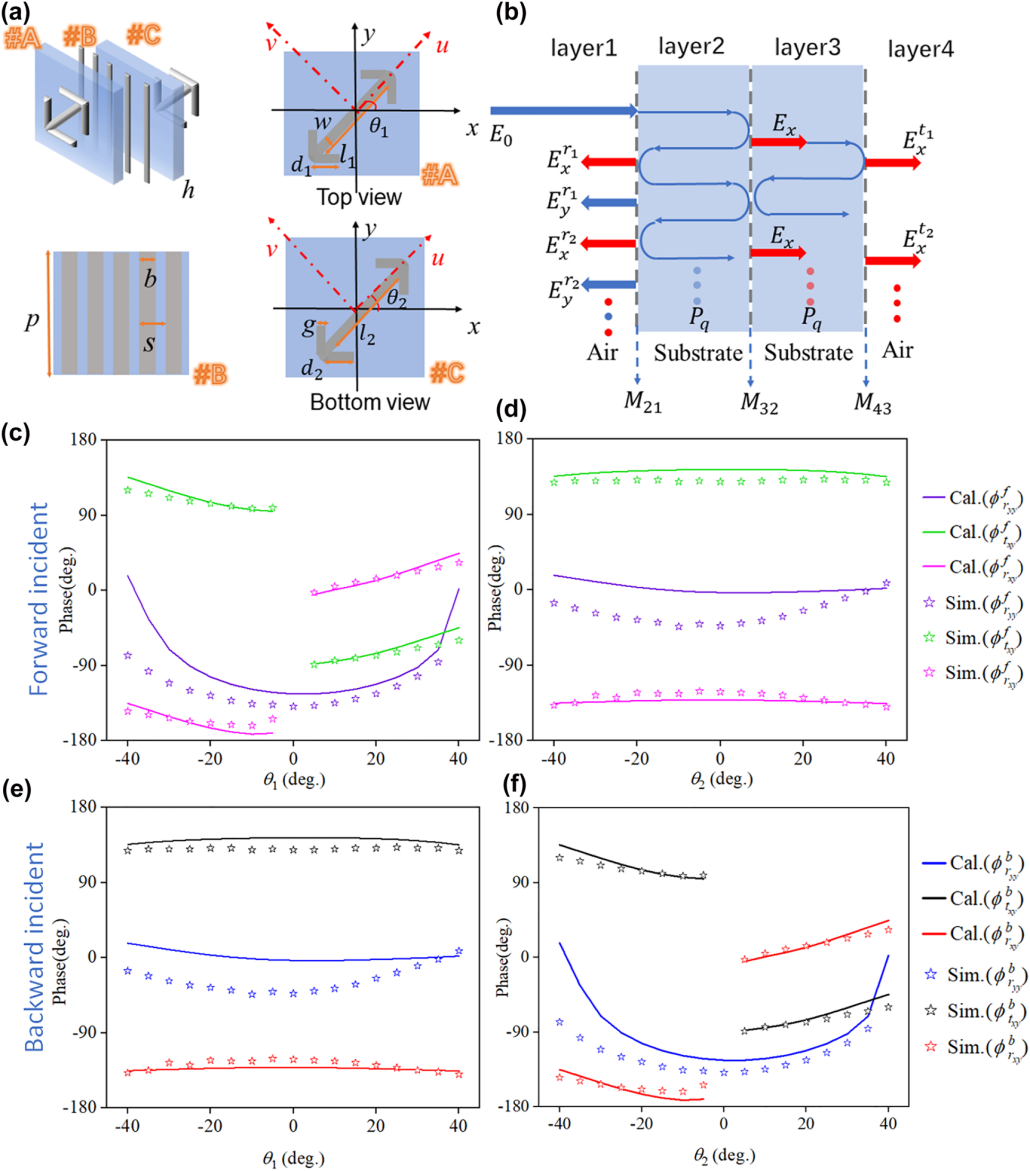
Design and numerical validation of the unit cell. (a) Schematic representation of the unit structure; on the right and below is an enlarged schematic diagram of structures #A, #B, and #C. (b) Schematics of the multiple reflection and transmission interference model in the three-layer metasurface. For *d*_1_ = 5 mm, *d*_2_ = 5 mm, *l*_1_ = 8 mm, *l*_2_ = 8 mm, the calculated and simulated phase at 10 GHz. (c) Forward reflection and transmission phase of the unit cell for varying angle *θ*_1_ with *θ*_2_ = −40°. (d) Forward reflection and transmission phase of the unit cell for varying angle *θ*_2_ with *θ*_1_ = −40°. (e) Backward reflection and transmission phase of the unit cell for varying angles *θ*_1_ with *θ*_2_ = −40°. (f) Backward reflection and transmission phase of the unit cell for varying angles *θ*_2_ with *θ*_1_ = −40°. *f* and *b* represent the incident wave from the front and back of the metasurface, respectively, $\phi_{r_{\sigma_{1}\;\sigma_{2}}}$ and $\phi_{t_{\sigma_{1}\;\sigma_{2}}}$ is the reflection and transmission phase of the *σ*_1_-polarization for a *σ*_2_-polarized incident wave.

First, we elaborate on the principle for our directional full-space scattering channels. Intuitively, polarization, direction and incident angle can be multiplexed degrees of freedom to load information, which, however, is challenging since it requires the independent control of multiple channels. Here, to tackle such a challenge, we tailor the stacked meta-atoms which have much more control parameters. Figure 2a shows the design schematics of the proposed metasurface implemented by bi-layered double-arrow-shaped structures (#A and #C) separated by a metal grating (#B). The elementary meta-atom is then composed of three-layer 18-μm-thick metallic planar structures and two intermediate dielectric layers (*ɛ*_r_ = 2.65, tan *δ* = 0.001 and *h* = 3 mm). The unit cell has the period *p* = 9 mm with the following geometric parameters, *b* = 0.6 mm, *s* = 1.8 mm, *w* = 0.4 mm, and *g* = 0.4 mm, which is constant throughout this work unless otherwise specified.

The topology configuration is adopted to be seen as a Fabry–Perot-like [50–52] cavity as shown in Figure 2b **.** To better illustrate the working principle, we analyze the full-space scattering characteristics using the transfer matrix method (TMM). When an incident wave is transmitted through the metasurface, a 4 × 4 transfer matrix *M*_*βα*_ is exploited to illustrate the relationship between the forward and backward propagating fields [52]:

(1) $$\left(\begin{array}{c} E_{\beta x}^{f}\\ E_{\beta y}^{f}\\ E_{\beta x}^{b}\\ E_{\beta y}^{b} \end{array}\right)=M_{\beta \alpha }\left(\begin{array}{c} E_{\alpha x}^{f}\\ E_{\alpha y}^{f}\\ E_{\alpha x}^{b}\\ E_{\alpha y}^{b} \end{array}\right)$$

where $E_{\alpha \sigma }^{f}$ and $E_{\beta \sigma }^{f}$ represent the electric field in the medium *α* and *β* with polarization *σ*(*σ* = *x*, *y*), and *f* and *b* indicate forward and backward propagating directions. *M*_*βα*_ is given as

(2) $$\begin{array}{cc} M_{\beta \alpha } & ={\left(\begin{array}{cccc} 1 & 0 & -r_{\beta x,\beta x} & -r_{\beta x,\beta y}\\ 0 & 1 & -r_{\beta y,\beta x} & -r_{\beta y,\beta y}\\ 0 & 0 & t_{\alpha x,\beta x} & t_{\alpha x,\beta y}\\ 0 & 0 & t_{\alpha y,\beta x} & t_{\alpha y,\beta y} \end{array}\right)}^{-1}\\ & \;\times \left(\begin{array}{cccc} t_{\beta x,\alpha x} & t_{\beta x,\alpha y} & 0 & 0\\ t_{\beta y,\alpha x} & t_{\beta y,\alpha y} & 0 & 0\\ -r_{\alpha x,\alpha x} & -r_{\alpha x,\alpha y} & 1 & 0\\ -r_{\alpha y,\alpha x} & -r_{\alpha y,\alpha y} & 0 & 1 \end{array}\right) \end{array}$$

where *r* and *t* are the reflection and the transmission coefficients, and the subscripts *βσ*, *ασ* denote that the light propagates from the medium *α* to *β* with polarization *σ*(*σ* = *x*, *y*). For the given homogeneous medium *ξ* with a refractive index *n*_*ξ*_(*ω*)*,* the propagation matrix is given by $P_{\xi }=\mathrm{diag}\left({\text{e}}^{\text{i}k_{0}n_{\xi }h},{\text{e}}^{\text{i}k_{0}n_{\xi }h},e^{-ik_{0}n_{\xi }h},e^{-ik_{0}n_{\xi }h}\right)$ , where *k*_0_ is the wave number in free-space. Finally, for a multilayered structure composed of several metamaterial and dielectric layers as shown in Figure 2b, the overall *M*-matrix can be written as [52].

(3) $$M=M_{43}P_{\xi }M_{32}P_{\xi }M_{21}$$

Following this derivation, the overall reflection matrix *R* and transmission matrix *T* can be obtained from Eqs. S5 and S6. Next, we discuss the transmission of different layers. For the linear polarization of the forward (along the -*z* direction) and normal incident layers #A and #C, the reflection and transmission characteristics can be described with the Jones matrix:

(4) $$R=\left(\begin{array}{cc} R_{xx} & R_{xy}\\ R_{yx} & R_{yy} \end{array}\right)$$

(5) $$T=\left(\begin{array}{cc} T_{xx} & T_{xy}\\ T_{yx} & T_{yy} \end{array}\right)$$

where $R_{\sigma_{1}\sigma_{2}}$ and $T_{\sigma_{1}\sigma_{2}}$ are the reflection and transmission coefficients of the *σ*_1_-polarization component under a *σ*_2_-polarized incident wave (*σ*_1_, *σ*_2_ = *x*, *y*). Due to the symmetrical feature of the proposed double-arrow-shaped structure, the polarization conversion efficiency of structures #A and #C will be suppressed to zero with *θ*_1,2_ = 0° (i.e. $R_{\sigma_{1}\sigma_{2}}=0$ , $T_{\sigma_{1}\sigma_{2}}=0$ (*σ*_1_ ≠ *σ*_2_)). When rotated with an angle of *θ*, the structure of the double-arrow-shaped structure will exhibit a reflection and a transmission Jones matrix: *R*(*θ*) = *A*(*θ*)^*T*^ ⋅ *R*(0) ⋅ *A*(*θ*), *T*(*θ*) = *A*(*θ*)^*T*^ ⋅ *T*(0) ⋅ *A*(*θ*), in which *A*(*θ*) represents the rotation matrix denoted by $A\left(\theta \right)=\left(\begin{array}{cc} \cos\theta & \sin\theta \\ -\sin\theta & \cos\theta \end{array}\right)$ . Then, we can obtain the matrices *M*_21_ and *M*_43_. In addition, the vertical metal grating structure (layer #B) can only transmit the *x*-polarized component and totally reflect the *y*-polarized wave, essential for splitting the transmission and reflection channels. When the *y*-polarized forward and normal incident to the layer #A. The transmitted *x-*polarized components can be expressed as: (which can be found from the Eq. S(10))

(6) $$E_{t,x}\left(\theta \right)=\frac{T_{xx}-T_{yy}}{2}\sin2\theta$$

which implies a positive correlation between the polarization conversion and the sine of the orientation angle for the certain *T*_*xx*_ and *T*_*yy*_. It is worthwhile to note that the reflected cross-polarization conversion has a similar expression. This result implies that the local phase keeps constant with the rotation angle *θ* ∈ [−*π*/2, 0), but undergoes a phase shift of *π* for *θ* ∈ [0, *π*/2). This method hence offers the controllable phase response by altering the rotation angle of the unit cell. Supposing the electric field of the incident wave expressed by *E*_0_, then the overall transmitted wave component can be deduced as [53]:

(7) $$\begin{array}{cc} E_{x}^{t} & =E_{x}^{t_{1}}+E_{x}^{t_{2}}+E_{x}^{t_{3}}+\cdot \cdot \cdot \\ & =t_{\mathrm{321}}t_{43}{\text{e}}^{\text{i}k_{3}h_{3}}E_{0}+t_{\mathrm{321}}t_{43}r_{\mathrm{123}}t_{43}e^{3ik_{3}h_{3}}E_{0}\\ & \;+t_{\mathrm{321}}t_{43}^{2}r_{\mathrm{123}}^{2}t_{43}e^{5ik_{3}h_{3}}E_{0}+\cdot \cdot \cdot \\ & =\frac{t_{\mathrm{321}}t_{43}{\text{e}}^{\text{i}k_{3}h_{3}}}{1-r_{\mathrm{123}}r_{43}e^{2ik_{3}h_{3}}}E_{0} \end{array}$$

where $t_{\mathrm{321}}=\frac{t_{21}t_{32}{\text{e}}^{\text{i}k_{2}h_{2}}}{1-r_{12}r_{32}e^{2ik_{2}h_{2}}}$ and $r_{\mathrm{123}}=r_{23}+\frac{t_{23}r_{12}t_{32}e^{2ik_{2}h_{2}}}{1-r_{12}r_{32}e^{2ik_{2}h_{2}}}$ . *r*_*ij*_ and *t*_*ij*_ denote the reflection and transmission coefficients from layer *j* to layer *i*(*i*, *j* = 1, 2, 3, 4), respectively. The propagation constant for each layer is expressed as *k*_*j*_ = 2*πη*_*j*_ *f*_1_/*c*, where *η*_*j*_ denotes the refractive index of layer *j*, *f*_1_ represents the frequency, *c* is the light velocity in air, and *h*_*j*_ is the thickness of the layer *j.*

We validate the function of the meta-atom by performing full-wave numerical simulations. We first consider the case of fixed parameters *d*_1_, *d*_2_, *l*_1_, *l*_2_ and *θ*_2_ while varying the rotation direction of *θ*_1_. For forward incidence as shown in Figure 2c, the phase of cross-polarized component ( $\phi_{t_{xy}}^{f}$ and $\phi_{r_{xy}}^{f}$ ) undergoes an abrupt *π* change, i.e. $\phi_{r_{xy}}^{f}\left(\theta_{1}\right)=\phi_{r_{xy}}^{f}\left(-\theta_{1}\right)\pm \pi$ , $\phi_{t_{xy}}^{f}\left(\theta_{1}\right)=\phi_{t_{xy}}^{f}\left(-\theta_{1}\right)\pm \pi$ , while the co-polarized component ( $\phi_{r_{yy}}^{f}$ ) remains the same, i.e. $\phi_{r_{yy}}^{f}\left(\theta_{1}\right)=\phi_{r_{yy}}^{f}\left(-\theta_{1}\right)$ . However, this does not change the phase responses for the backward *y*-polarized incidence ( $\phi_{t_{xy}}^{b}$ , $\phi_{r_{xy}}^{b}$ and $\phi_{r_{yy}}^{b}$ ), as illustrated in Figure 2e. When parameters *d*_1_, *d*_2_, *l*_1_, *l*_2_ and *θ*_1_ are fixed while varying *θ*_2_, a similar conclusion can be deduced. As shown in Figure 2d and f, the phase change of *π* change can be easily introduced for $\phi_{t_{xy}}^{b}$ and $\phi_{r_{xy}}^{b}$ (e.g. $\phi_{r_{xy}}^{b}\left(\theta_{2}\right)=\phi_{r_{xy}}^{b}\left(-\theta_{2}\right)\pm \pi$ , $\phi_{t_{xy}}^{b}\left(\theta_{2}\right)=\phi_{t_{xy}}^{b}\left(-\theta_{2}\right)\pm \pi$ ), and the co-polarized component ( $\phi_{r_{yy}}^{b}$ ) remains the same, i.e. $\phi_{r_{yy}}^{b}\left(\theta_{2}\right)=\phi_{r_{yy}}^{b}\left(-\theta_{2}\right)$ , the effect of changing phase ( $\phi_{t_{xy}}^{f}$ , $\phi_{r_{xy}}^{f}$ and $\phi_{r_{yy}}^{f}$ ) is negligible when changing *θ*_2_. In addition, for the *y*-polarized forward propagating wave, the parameters *d*_2_ and *l*_2_ of the layer #C show relatively little influence on $\phi_{r_{yy}}^{f}$ , $\phi_{t_{xy}}^{f}$ and $\phi_{r_{xy}}^{f}$ , which can be obtained as described in Figure S1 in the Text S1 of the Supplementary Information. The calculation results are in good agreement with the simulation results, which can be found in Figure 2c–f and Figure S2 in the Supplementary Information. Hence, for forward (backward) wave incidences, the desired phase of reflection and transmission components should mainly rely on geometric parameters and rotation angle of structure #A (#C). Such intriguing isolation is the key point for engineering asymmetric bidirectional multiplexing functions. Unlike unit cells with cascading anisotropic impedance sheets in a twisted form [54], where unidirectional transmission properties come from two opposite directions, our proposed meta-atoms operate under both propagating directions with independent phase control properties, making our proposed Janus metasurface with non-interleaved assignment possible. Hence, this design suggests that the broken mirror symmetry for forward and backward transmission can support the independent control of the multi-channel scattering, which is very promising for compact devices.

## 3 Spatial symmetry breaking for directional reflection-transmission multitasking meta-holograms

Systems that implement different functions in opposite directions have considerable potential in radar, communications, and multiple-input multiple-output systems. The above research on the unit structure provides powerful enlightenment for multitask reflection-transmission wave control. It is worth noting that a complete parametric study on geometrical parameters *d*_1_, *d*_2_, *l*_1_, *l*_2_, *θ*_1_ and *θ*_2_ of such Janus metasurface can further provide possibilities for the realization and utilization of the co-polarized reflection and cross-polarized reflection and transmission holograms. The coding unit cells are exhibited at the output of this process, exposing arbitrary 1-bit phases of “0” and “*π*” in each channel, which can be digitally represented by

(8) $$K_{1-bit}=\left(\begin{array}{cccc} 0/0/0/0 & 0/0/0/1 & 0/0/1/0 & 0/0/1/1\\ 0/1/0/0 & 0/1/0/1 & 0/1/1/0 & 0/1/1/1\\ 1/0/0/0 & 1/0/0/1 & 1/0/1/0 & 1/0/1/1\\ 1/1/0/0 & 1/1/0/1 & 1/1/1/0 & 1/1/1/1 \end{array}\right)$$

**Figure 3:**
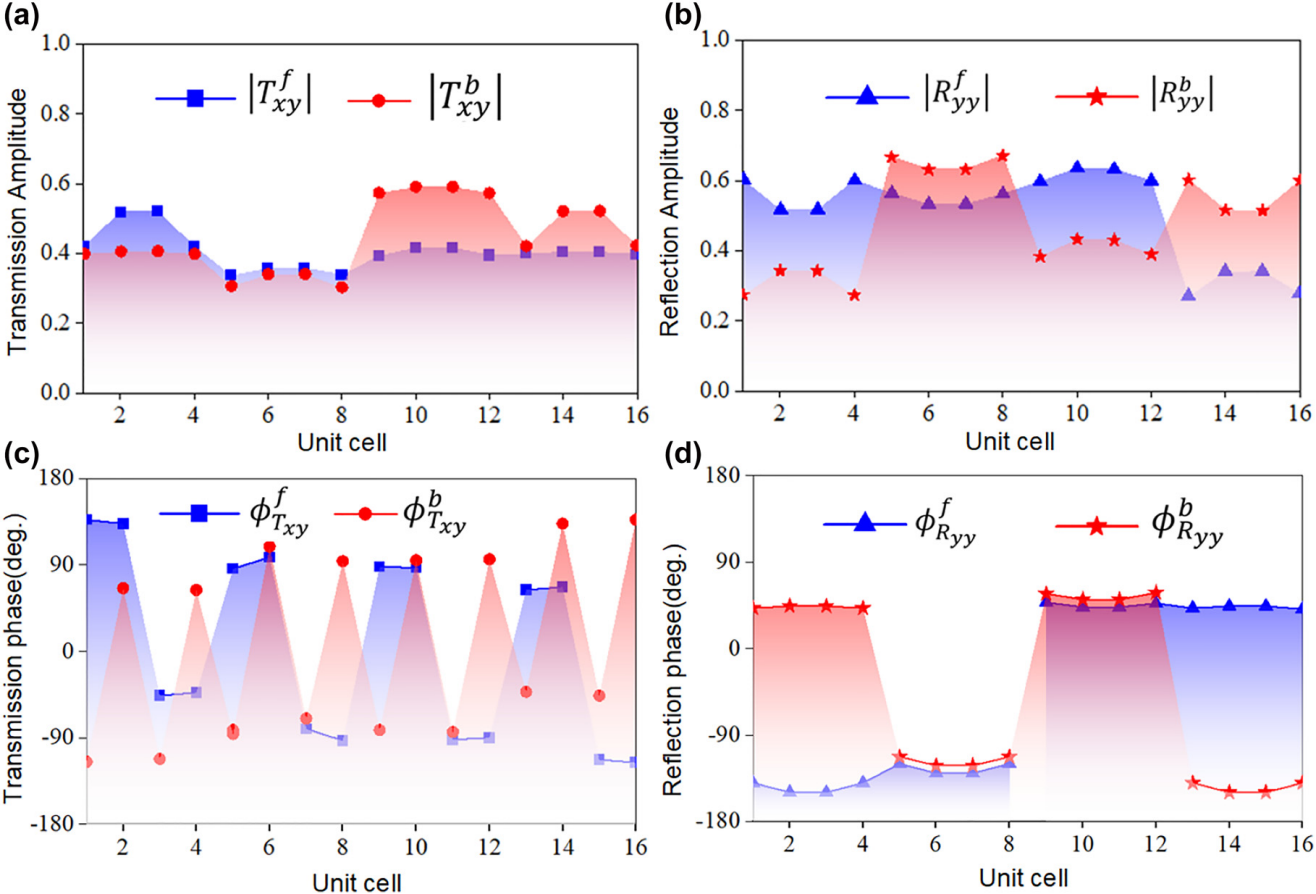
Characterization of the composite meta-atoms for reflection and transmission multitasking under *y*-polarized forward and backward illuminations. (a) Cross-polarized transmission and (b) co-polarized reflection amplitude responses. (c) Cross-polarized transmission and (d) co-polarized reflection phase responses.

Here, the numbers separated by the slash sign represent polarizations states of reflection and transmission when a *y*-polarized wave is incident in the forward and backward direction, i.e. *R* ^*f*^_*yy*_/*R* ^*b*^_*yy*_/*T* ^*f*^_*xy*_/*T* ^*b*^_*xy*_, respectively. Significantly, the sign of the phase change can offer flexibility to develop multiple functionalities under oppositely directed incident wave illuminations. The meticulously optimized construction parameters are presented in Table S1 (see Text S2 of Supplementary Information for details). The cross-polarized transmission and co-polarized reflection amplitudes are plotted in Figure 3a and b **,** and the corresponding phase distributions are illustrated in Figure 3c and d under forward and backward incidence. It can be seen that under *y*-polarized forward or backward incident wave illumination, the phase of both directional reflection and transmission present two types of state, with a difference of *π*, guaranteeing the conversion of reflection and transmission waves. Such more degree of freedom affords us to further demonstrate different functions in the reflection and transmission spaces for opposite incident wave directions. Two applications are selected as examples to verify our proposal: one is to focus energy at different spots, and another one is holography with different images according to the transmission or reflection mode from opposite illumination directions.

**Figure 4:**
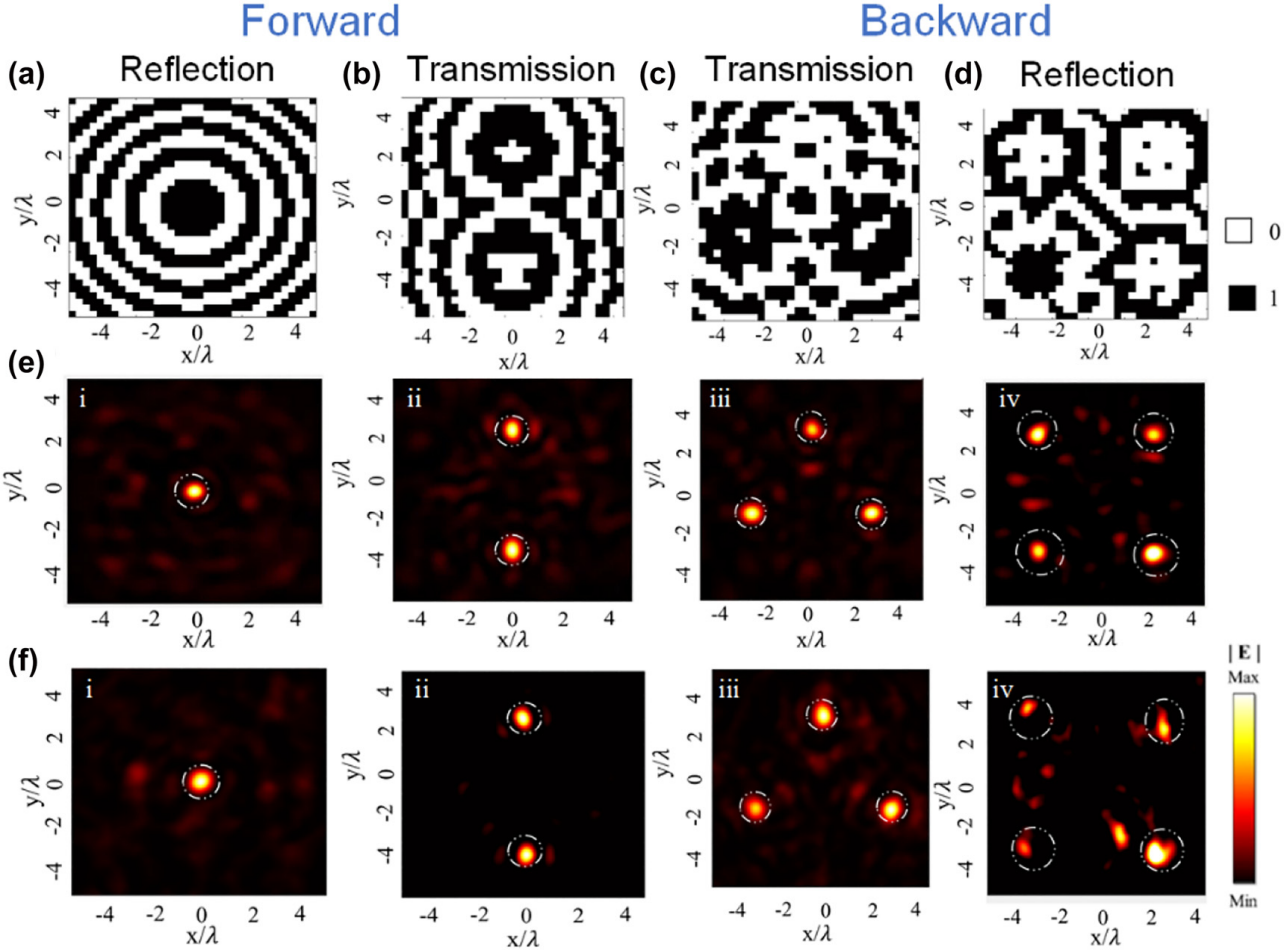
Characterization for the reflection and transmission multichannel focusing metasurface. The metasurface with overall dimensions of 279 × 279 mm^2^ in the *xoy* plane is composed of 31 × 31 unit cells. (a) and (b) Phase distribution of the co-polarized reflected and cross-polarized transmitted channels for the linearly polarized forward incident wave. (c) and (d) Phase distribution of the cross-polarized transmitted and co-polarized reflected channels for linearly polarized backward incident wave. (e) Electric field distribution of corresponding simulation results in each channel. (f) Electric field distributions of corresponding experimental results in each channel.

First, we demonstrate a metasurface with focusing functionalities, which integrates reflection to one/four spots and transmission to two/three spots under the *y*-polarized forward/backward incidence. More importantly, the forward and backward scatting channels of co-polarized reflection and cross-polarized transmission can be independently modulated by using all unit cells. The ideal phase for the different focusing configurations is calculated and shown in Figure 4a–d. For forward incidence, the co-polarized reflected wave is focused to one spot and the cross-polarized reflected and transmitted waves are focused to two spots. However, for backward direction, the co-polarized reflected wave is focused to four spots and the cross-polarized reflected and transmitted waves are focused to three spots. As illustrated in Figure 4e and f **,** the measured results are in line with the simulations, promising further development of multiple functionalities. In addition, the amplitude and phase responses of the cross-polarized reflection are described in Figure S3 of Text S3 in the Supplementary Information, and the corresponding focusing features can be found in the Figure S4 in the Supplementary Information. The reflection/transmission efficiency, which expounds the reflection/transmission characteristic of the proposed metasurface, is defined as the ratio of the total intensity of the wave in each specific channel to the incident intensity on a surface of the same size. Under *y*-polarized forward incidence, the co-polarized reflection, cross-polarized reflection and cross-polarized transmission efficiency is calculated to be 28.6%, 23.67%, 7.77%, and the total energy utilization is 59.74%. The efficiency of each channel corresponding to the backward incident is 23.05%, 21.07%, 10.93%, which means the total energy utilization is 55.05%. The relatively low energy of the transmission channel is mainly due to the large losses attributed to the resonant nature of the unit cells.

**Figure 5:**
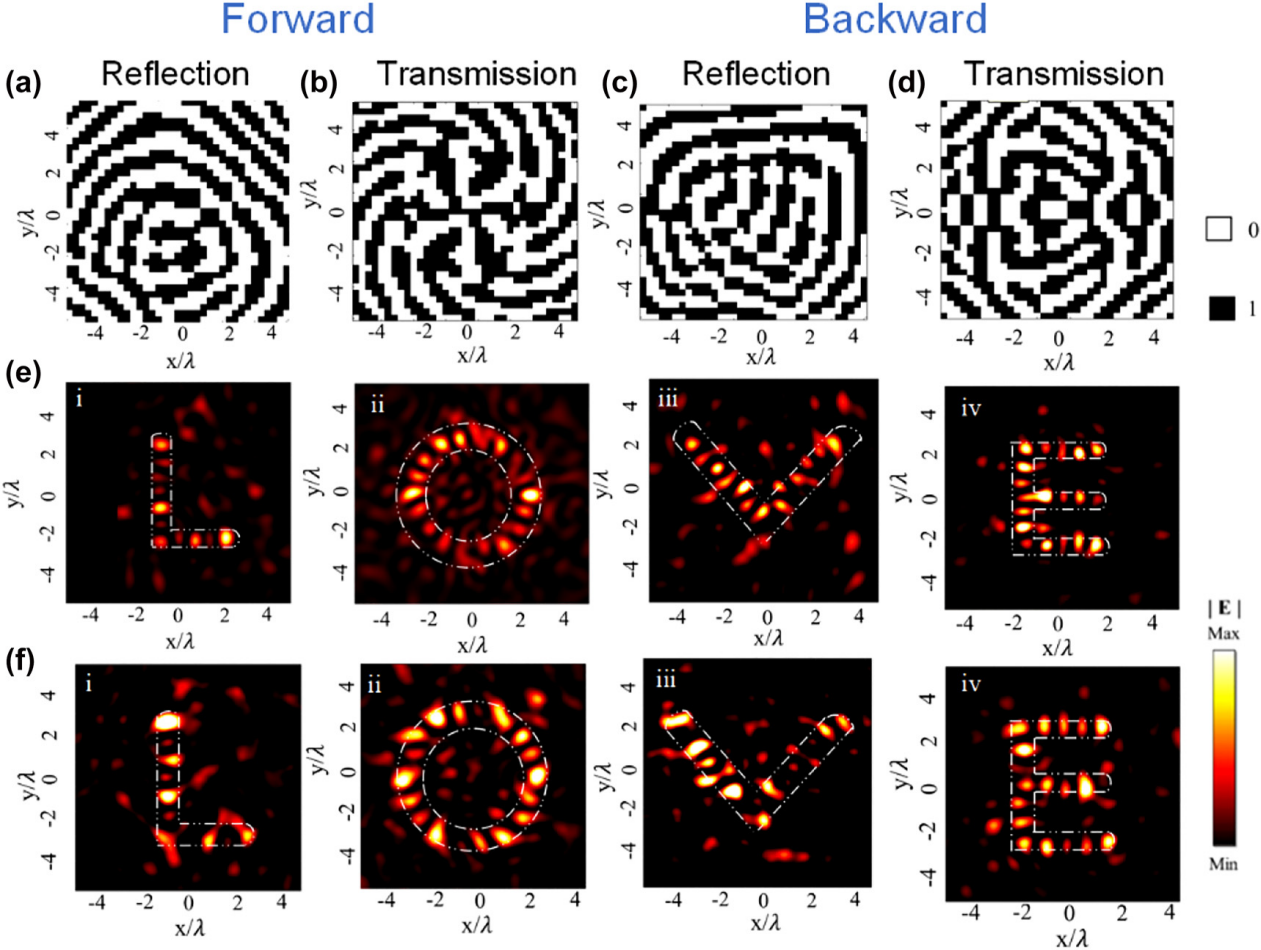
Characterization for the reflection-transmission-integrated multichannel holographic metasurface. (a) and (b) Phase distribution of the co-polarized reflected image “L” and cross-polarized transmitted image “O” with the linearly polarized forward incident wave. (c) and (d) Phase distribution of the co-polarized reflected image “V” and cross-polarized transmitted image “E” with linearly polarized backward incident wave. (e) Electric field distribution of corresponding simulation results in each channel. (f) Electric field distributions of corresponding experimental results in each channel.

Holography has become an attractive technology because of its characteristics for recording and reconstructing object images by storing and releasing phase information. Traditional methods have limitations such as bulky volumetric occupation and narrow spatial bandwidth. In contrast, metasurface holography can provide unprecedented resolution, low noise, and high-precision reconstructed images, which can be beneficial to short-range communication systems, detection, security, data storage and information processing [55–64]. To validate the powerful ability of generating different holograms in the two semi-spaces under oppositely directed incident illuminations, we propose another advanced feature metasurface composed of 35 × 35 meta-units with the size of 315 × 315 mm^2^, which is fabricated using common printed circuit board (PCB) technique (see Text S4 of Supplementary Information for details). As schematically shown in Figure 1c, under the forward illumination of the *y*-polarized wave, a co-polarized reflected image “L” and a cross-polarized transmitted image “O” can be produced. A co-polarized reflected image “V” and a cross-polarized transmitted image “E” are generated with the backward illumination of the *y*-polarized wave at 10 GHz. The distance between the reflection/transmission plane and the metasurface is set as 80 mm. The modified weighted Gerchberg–Saxton (GSW) algorithm is adopted to obtain the desired phase distribution, and the coding patterns are shown in Figure 5a–d. Interestingly, both reflected and transmitted images in opposite regions are different, validating the concept of generating independent holographic images with non-interleaved metasurfaces. This cannot be realized by traditional holographic devices. As illustrated in Figure 5e and f, the numerical and measurement results are in concordance with each other, exhibiting four alphabet letters. Since the “LOVE” image in those channels (co-polarized reflection and cross-polarized transmission) are optimized by the algorithm respectively and that the crosstalk between reconstructed images can be ignored, it can be imagined that arbitrary hologram with directional features can be accomplished by the proposed transmission-reflection-integrated Janus metasurface. The cross-polarized reflection hologram performances can be found in Figure S5 in the Supplementary Information. The imaging efficiency, defined as the ratio of energy in focal points to the total transmitted energy in the measurement plane, is calculated as 62.3%, 64.3%, 68.4% for the co-polarized reflection (“L”), cross-polarized reflection (“O”) and cross-polarized transmission (“O”) channels for the forward incidence, and 61.5%, 63.65% and 67.6% for the images “V”, “E” and “E” for the backward incidence, respectively. Fabrication and measurement tolerances are the two factors that have an impact on the imaging efficiency. Slight change of dielectric constant caused by the dispersion characteristics of the dielectric plate and the cured sheet introduced in the processing of the multilayer plate can influence the imaging performances. Additionally, placement and slight misalignment of samples during measurement of the prototype and influence of cables can also be a source of losses. It should be emphasized that the metasurface is passive and arranged with a non-interleaved scheme, where each meta-atom plays full role in both reflection and transmission channels under oppositely directed incident illuminations.

## 4 Conclusions

Fascinating phenomena appear in the transmission-reflection integrated multitask Janus metasurface, enabling a new degree of the manipulation of the electromagnetic waves in the opposite directions. Further work can be dedicated to extend the bandwidth of the devices and to transpose the proposed concept to millimeter and terahertz wave. In addition, we can even resort to more scattering degrees of freedom by introducing *x*-polarized waves incidents in different directions, which can further improve the multifunctional capacity of the metasurface.

In summary, we have proposed and demonstrated a novel transmission-reflection-integrated multitask Janus metasurface. The encoding metasurface enables simultaneous control of the opposite directional propagating wave with distinct functions only by introducing meta-atom designs with non-interleaved arrangement at microwave frequencies. Different channels can be switched by setting different optical paths, and all images are displayed independently. The advanced functionality realized for holograms in two directions goes far beyond previous attempts for unidirectional functions. A series of calculated and experimental results demonstrate that our asymmetric approach simultaneously fulfills completely independent full space imaging for both forward and backward propagation directions. Our demonstrated transmission-reflection-integrated multifunctional Janus metasurface may facilitate advanced compact-imaging system and space multiplexed, chiral sensing, data encryption and decryption and other applications.

## Supplementary Material

Supplementary Material Details
