# Supplementary material for: A non-interleaved bidirectional Janus metasurface with full-space scattering channels
Source: Nanophotonics. 2022 Aug 5;11(16):3729–39. doi: 10.1515/nanoph-2022-0292 (PMC11501967; doi:10.1515/nanoph-2022-0292)
Supplement: Supplementary file 1 — Supplementary Material Details [file j_nanoph-2022-0292_suppl.docx]

Supporting Information

Title: A Non-Interleaved Bidirectional Janus Metasurface with Full-Space Scattering Channels

Guanyu Shang^a^, Guangwei Hu^a^, Chunsheng Guan^a^, Yue Wang, Kuang Zhang, Qun Wu, Jian Liu, Xue-Mei Ding, Shah Nawaz Burokur^*^, Haoyu Li^*^, Xumin Ding^*^ and Cheng-Wei Qiu^*^

^a^ **Guanyu Shang, Guangwei Hu, Chunsheng Guan**: These authors contributed equally to this work

^*^Corresponding authors: **Cheng-wei Qiu**, Department of Electrical and Computer Engineering, National University of Singapore, Singapore 117583, Singa-pore, e-mail: [chengwei.qiu@nus.edu.sg](mailto:chengwei.qiu@nus.edu.sg);

**Xumin Ding**, Advanced Microscopy and Instrumentation Research Center, Harbin Institute of Technology, Harbin 150080, China, e-mail: xuminding@hit.edu.cn;

**Haoyu Li**, State Key Laboratory of Robotics and Systems, Harbin Institute of Technology, Harbin 150080, China; Advanced Microscopy and Instrumentation Research Center, Harbin Institute of Technology, Harbin 150080, China, e-mail: lihaoyu@hit.edu.cn;

**Shah Nawaz Burokur**, LEME, UPL, Univ Paris Nanterre, F92410 Ville d’Avray, France, e-mail: sburokur@parisnanterre.fr;

**Guanyu Shang, Jian Liu,** and **Xuemei Ding**: Advanced Microscopy and Instrumentation Research Center, Harbin Institute of Technology, Harbin 150080, China

**Guangwei Hu**: Department of Electrical and Computer Engineering, National University of Singapore, Singapore 117583, Singa-pore

**Chunsheng Guan, Yue Wang, Kuang Zhang** and **Qun Wu**: Department of Microwave Engineering, Harbin Institute of Technology, Harbin 150001, China

**Text S1.** **Design principle of meta-atom.**

The mechanism of the asymmetric circular polarizer can be analyzed by the transfer matrix method. When an incident wave is transmitted through the metasurface, a 4 × 4 matrix *M_βα_* can be used to relate the forward to the backward propagating fields [52]

$\left(\begin{matrix} E_{\beta x}^{f} \\ E_{\beta y}^{f} \\ E_{\beta x}^{b} \\ E_{\beta y}^{b} \end{matrix}\right)=M_{\beta\alpha}\left(\begin{matrix} E_{\alpha x}^{f} \\ E_{\alpha y}^{f} \\ E_{\alpha x}^{b} \\ E_{\alpha y}^{b} \end{matrix}\right)$ (S1)

where $E_{\alpha\sigma}^{f}$ and $E_{\beta\sigma}^{f}$ represents the electric field in the medium *α* and *β* with polarization $\sigma(\sigma=x,y)$, and *f* and *b* indicate forward and backward propagating directions. *M_βα_* can be depicted as

$M_{\beta\alpha}\text{=}\left(\begin{matrix} 1 & 0 & -r_{\beta x,\beta x} & -r_{\beta x,\beta y} \\ 0 & 1 & -r_{\beta y,\beta x} & -r_{\beta y,\beta y} \\ 0 & 0 & t_{\alpha x,\beta x} & t_{\alpha x,\beta y} \\ 0 & 0 & t_{\alpha y,\beta x} & t_{\alpha y,\beta y} \end{matrix}\right)^{-1}\times\left(\begin{matrix} t_{\beta x,\alpha x} & t_{\beta x,\alpha y} & 0 & 0 \\ t_{\beta y,\alpha x} & t_{\beta y,\alpha y} & 0 & 0 \\ -r_{\alpha x,\alpha x} & -r_{\alpha x,\alpha y} & 1 & 0 \\ -r_{\alpha y,\alpha x} & -r_{\alpha y,\alpha y} & 0 & 1 \end{matrix}\right)$ (S2)

where *r* and *t* are the reflection and the transmission coefficients, and the subscripts $\beta\sigma,\alpha\sigma$ denotes that the light is incident from the medium *α * to *β* with polarization $\sigma(\sigma=x,y)$. For the given homogeneous medium $\xi$ with refractive index $n_{\xi}(\omega)$*,* the propagation matrix is given by $P_{\xi}=diag(e^{ik_{0}n_{\xi}h},e^{ik_{0}n_{\xi}h},e^{-ik_{0}n_{\xi}h},e^{-ik_{0}n_{\xi}h})$, where *k*_0_ is the wave number in free-space. Finally, for a structure composed of several metamaterial and dielectric layers as shown in Figure 2b, the overall *M*-matrix can be written as [52]

$M=M_{43}P_{\xi}M_{32}P_{\xi}M_{21}$ (S3)

The *M*-matrix simplifies to:

$M=\left(\begin{matrix} \left(\begin{matrix} m_{11} & m_{12} \\ m_{21} & m_{22} \end{matrix}\right) & \left(\begin{matrix} m_{13} & m_{14} \\ m_{23} & m_{24} \end{matrix}\right) \\ \left(\begin{matrix} m_{31} & m_{32} \\ m_{41} & m_{42} \end{matrix}\right) & \left(\begin{matrix} m_{33} & m_{34} \\ m_{43} & m_{44} \end{matrix}\right) \end{matrix}\right)$ (S4)

Then, the overall reflection matrix *R* and transmission matrix *T* can be obtained as

$R_{total}=-\left(\begin{matrix} m_{33} & m_{34} \\ m_{43} & m_{44} \end{matrix}\right)^{-1}\left(\begin{matrix} m_{31} & m_{32} \\ m_{41} & m_{42} \end{matrix}\right)$ (S5)

$T_{total}=\left(\begin{matrix} m_{11} & m_{12} \\ m_{21} & m_{22} \end{matrix}\right)-\left(\begin{matrix} m_{13} & m_{14} \\ m_{23} & m_{24} \end{matrix}\right)\left(\begin{matrix} m_{33} & m_{34} \\ m_{43} & m_{44} \end{matrix}\right)^{-1}\left(\begin{matrix} m_{31} & m_{32} \\ m_{41} & m_{42} \end{matrix}\right)$ (S6)

Finally, the overall reflection and transmission coefficients can be retrieved from the matrix *M*. Next, we discuss the transmission of different layers. For the linear polarization of the forward (along the -*z* direction) and normal incident layer #A and #C, the reflection and transmission characteristics can be described with the Jones matrix:

$R=\left(\begin{matrix} R_{xx} & R_{xy} \\ R_{yx} & R_{yy} \end{matrix}\right)$ (S7)

$T=\left(\begin{matrix} T_{xx} & T_{xy} \\ T_{yx} & T_{yy} \end{matrix}\right)$ (S8)

where $R_{\sigma_{1}\sigma_{2}}$ and $T_{\sigma_{1}\sigma_{2}}$ are the reflection and transmission coefficients of the $\sigma_{1}$-polarization component under a $\sigma_{2}$-polarized incident wave $(\sigma_{1},\sigma_{2}=x,y)$. Due to the symmetrical feature of the proposed double-arrow-shaped structure, the polarization conversion efficiency of structures #A and #C will be suppressed to zero with $\theta_{1,2}=0^{\circ}$(*i.e.* $R_{\sigma_{1}\sigma_{2}}=0$, $T_{\sigma_{1}\sigma_{2}}=0$ $(\sigma_{1}\neq\sigma_{2})$). If rotated with an orientation $\theta$, the structure of the double-arrow-shaped structure will exhibit a reflection and a transmission Jones matrix:$R(\theta)=A(\theta)^{T}\cdot R(0)\cdot A(\theta)$，$T(\theta)=A(\theta)^{T}\cdot T(0)\cdot A(\theta)$，in which $A(\theta)$represents the rotation matrix denoted by $A(\theta)=\left(\begin{matrix} \cos\theta& \sin\theta\\ -\sin\theta& \cos\theta\end{matrix}\right)$.With the expansion calculation, the reflection and the transmission Jones matrix:

$\begin{matrix} R(\theta)=\left(\begin{matrix} \cos\theta& -\sin\theta\\ \sin\theta& \cos\theta\end{matrix}\right)\left(\begin{matrix} R_{xx} & 0 \\ 0 & R_{yy} \end{matrix}\right)\left(\begin{matrix} \cos\theta& \sin\theta\\ -\sin\theta& \cos\theta\end{matrix}\right) \\ \begin{matrix} & \end{matrix}=\left(\begin{matrix} R_{xx}\cos^{2}\theta+R_{yy}\sin^{2}\theta& R_{xx}\sin\theta\cos\theta-R_{yy}\sin\theta\cos\theta\\ R_{xx}\sin\theta\cos\theta-R_{yy}\sin\theta\cos\theta& T_{yy}\cos^{2}\theta+T_{xx}\sin^{2}\theta\end{matrix}\right) \end{matrix}$ (S9)

$\begin{matrix} T(\theta)=\left(\begin{matrix} \cos\theta& -\sin\theta\\ \sin\theta& \cos\theta\end{matrix}\right)\left(\begin{matrix} T_{xx} & 0 \\ 0 & T_{yy} \end{matrix}\right)\left(\begin{matrix} \cos\theta& \sin\theta\\ -\sin\theta& \cos\theta\end{matrix}\right) \\ \begin{matrix} & \end{matrix}=\left(\begin{matrix} T_{xx}\cos^{2}\theta+T_{yy}\sin^{2}\theta& T_{xx}\sin\theta\cos\theta-T_{yy}\sin\theta\cos\theta\\ T_{xx}\sin\theta\cos\theta-T_{yy}\sin\theta\cos\theta& T_{yy}\cos^{2}\theta+T_{xx}\sin^{2}\theta\end{matrix}\right) \end{matrix}$ （S10）

Through the above analysis, we can get the matrix $M_{21}$and $M_{43}$. From the equation S10, when the *y*-polarized forward and normal incident to the layer #A. The transmitted *x-*polarized components can be expressed as:

$\vec{E}_{t,x}(\theta)=\frac{T_{xx}-T_{yy}}{2}\sin2\theta$ (S11)

which implies a positive correlation between the polarization conversion and the sine of the orientation angle for the certain $T_{xx}$ and $T_{yy}$. The reflected cross-polarization conversion has a similar expression. This result implies that the local phase keeps constant with the rotation angle $\theta\in[-\pi/2,0)$, but undergoes a phase shift of π for $\theta\in[0\text{，}\pi/2)$. This method hence offers the controllable phase response by altering the rotation angle of the unit cell. In addition, due to the vertical metal grating structure as the middle layer structure #B, only the *x*-polarized wave can be transmitted, but the *y*-polarized wave will be totally reflected, meaning that the only waves that are going into layer #C are *x*-polarized waves. When the x-polarized wave is incident to the layer #C, the transmitted co-polarized components can be expressed as

$\vec{E}_{t,x}(\theta)=T_{xx}\cos^{2}\theta+T_{yy}\sin^{2}\theta$ （S12）

The angle of #C has little effect on x-polarization, and this view is verified by experiments. In order to further verify the influence of parameter *d*_2_ and *l*_2_ changes in the layer (#C) on the forward incident electromagnetic wave, the simulation results through different parameters are shown in Figure S1. Varying *d*_2_ and *l*_2_ has little influence on forward co-polarized reflection, cross-polarized reflection and transmission phases of the meta-atom. The influence of the angle of the layer (#C) on the reflection and transmission channels of forward incidence is described in the text. Similarly, for the backward incident polarization wave, the control of the corresponding channel is mainly the parameter control of the structure layer (#A). So the asymmetric bidirectional Janus functionalities are engineered with direction-encoded distinct transmission phase patterns realized by modifying the parameters of structures #A and #C.


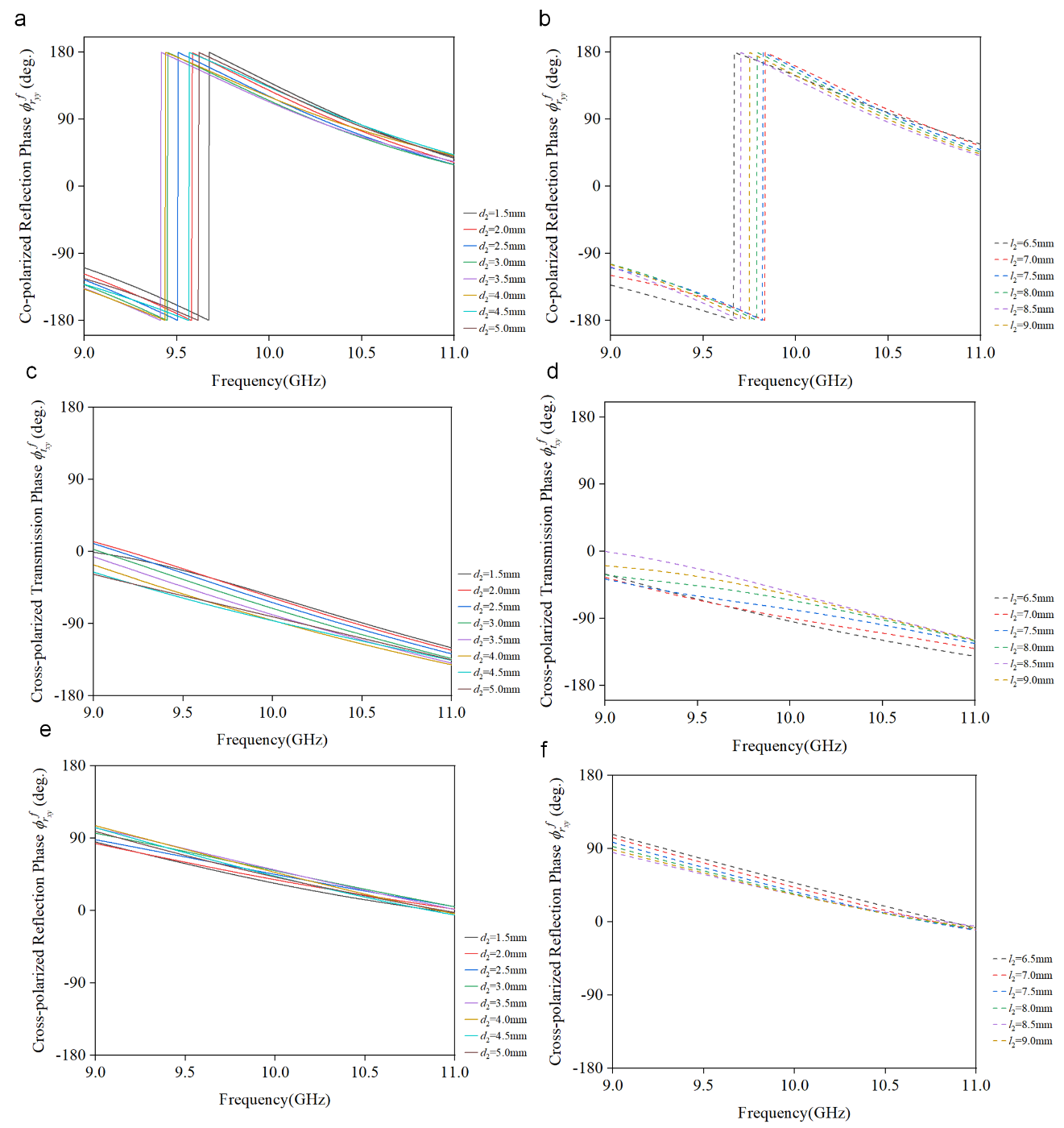


**Figure S1** Influence of parameter change on the phase of each channel. (a) and (b) Forward co-polarized reflection phase of the meta-atom with varying d_2_ and *l*_2_. (c) and (d) Forward cross-polarized transmission phase of the meta-atom with varying d_2_ and *l*_2_. (e) and (f) Forward cross-polarized reflection phase of the meta-atom with varying d_2_ and *l*_2_.

We validate the function of the meta-atom by performing full-wave numerical simulations (CST Microwave Studio). As shown in Figure S2, the calculated co-polarized reflection and cross-polarized reflection and transmission spectra agree well with the simulation ones with different angles. It can be found that the amplitude of co-polarization reflection decreases with the increase of angle, while the channel of cross-polarization reflection and transmission increases with the increase of angle. Therefore, during the design of the unit structure, the corresponding angles are optimized so that the energies in the three channels are as same as possible.


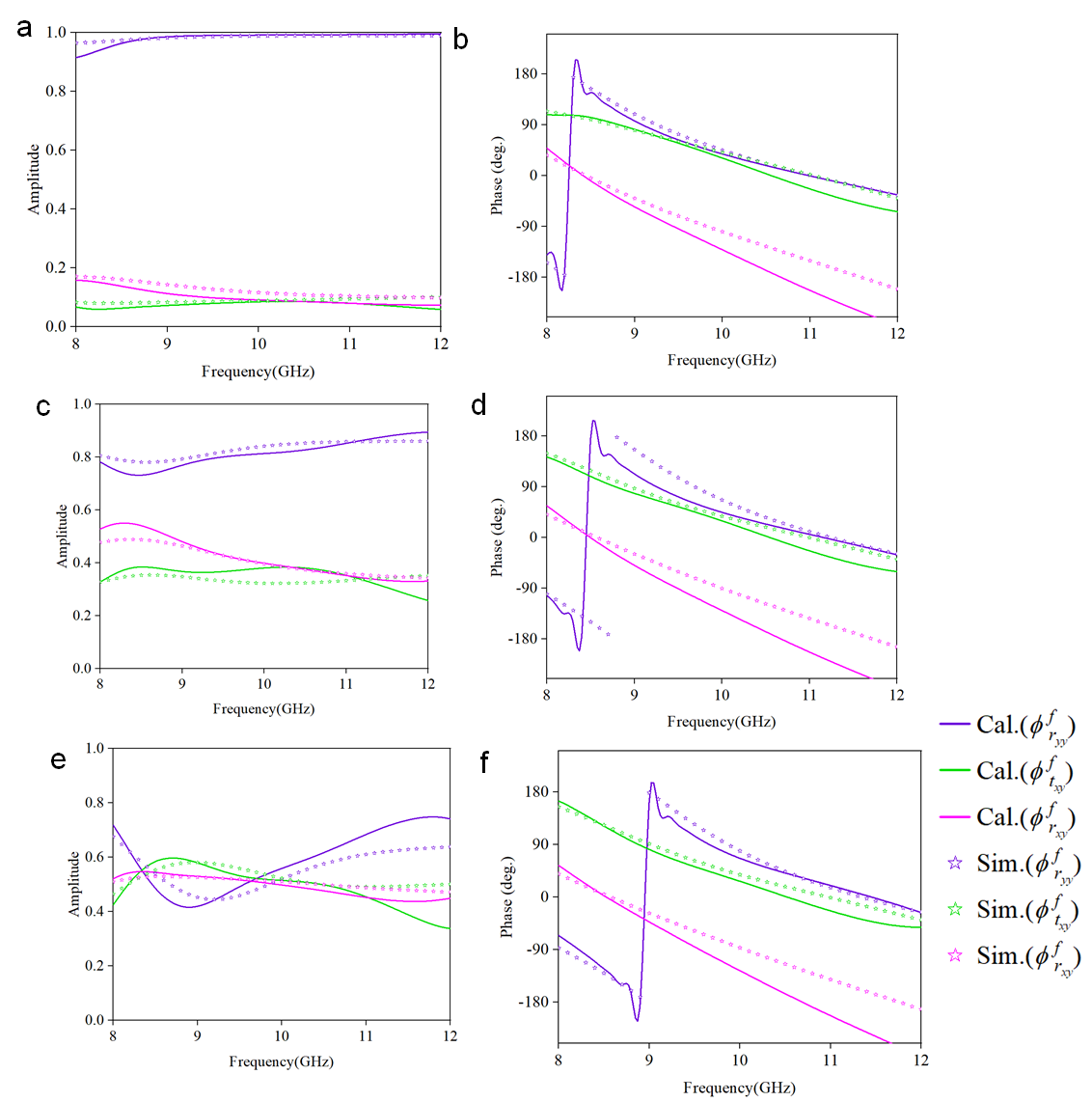


**Figure S2** Comparison between the calculated results from the transfer matrix method (TMM) and simulated amplitude and phase of the unit cell with *d*_1_ = 5 mm, *d*_2_ = 5 mm, *l*_1_ = 8 mm, *l*_2_ =8 mm, (a) and (b) Co-polarized reflection amplitude and phase of the meta-atom with *θ*_1_=*θ*_2_=5^0^. (c) and (d) Cross-polarized transmission amplitude and phase of the meta-atom with *θ*_1_=*θ*_2_=25^0^. (e) and (f) Cross-polarized reflection amplitude and phase of the meta-atom with *θ*_1_=*θ*_2_=40^0^.

**Text S2. Construction parameters of the Transmission-reflection integrated multitask meta-atoms.**

To realize the desired metasurface with different functionalities in reflection and transmission under different incident directions, the co-polarized reflection is controlled by ingeniously designing the parameters of the unit cell. For the sake of demonstration, 16 meta-atoms are wisely designed and optimized by adjusting changes in the following parameters, as detailed in Table S1.

**Table S1**. Construction parameters and corresponding phase of the coding meta-atoms.

| Coding  meta-atom | Construction parameters | | | | | |
| --- | --- | --- | --- | --- | --- | --- |
|  | $d_{1}$(mm) | $l_{1}$(mm) | $d_{2}$(mm) | $l_{2}$(mm) | $\theta_{1}$(deg) | $\theta_{2}$(deg) |
| 0/0/0/0 | 1 | 7 | 5 | 9 | -35 | -35 |
| 0/0/0/1 | 1 | 7 | 5 | 9 | -35 | 35 |
| 0/0/1/0 | 1 | 7 | 5 | 9 | 35 | -35 |
| 0/0/1/1 | 1 | 7 | 5 | 9 | 35 | 35 |
| 0/1/0/0 | 1 | 7 | 1 | 7 | -35 | 30 |
| 0/1/0/1 | 1 | 7 | 1 | 7 | -35 | -30 |
| 0/1/1/0 | 1 | 7 | 1 | 7 | 35 | 30 |
| 0/1/1/1 | 1 | 7 | 1 | 7 | 35 | 30 |
| 1/0/0/0 | 5 | 9 | 5 | 9 | 30 | 40 |
| 1/0/0/1 | 5 | 9 | 5 | 9 | 30 | -40 |
| 1/0/1/0 | 5 | 9 | 5 | 9 | -30 | -40 |
| 1/0/1/1 | 5 | 9 | 5 | 9 | -30 | 40 |
| 1/1/0/0 | 5 | 9 | 1 | 7 | 35 | 35 |
| 1/1/0/1 | 5 | 9 | 1 | 7 | -35 | 35 |
| 1/1/1/0 | 5 | 9 | 1 | 7 | -35 | 35 |
| 1/1/1/1 | 5 | 9 | 1 | 7 | -35 | -35 |

**Text S3.** **Cross-polarized reflection channels of the Transmission-reflection integrated multitask Janus metasurface with bidirectional functions.**

The phase and amplitude responses of the co-polarized reflected and cross-polarized transmitted waves for bi-directional incidence have been given in the main text of the paper. Here, we present comprehensive results of the cross-polarized reflection to further demonstrate our declaration. The simulated amplitude and phase profiles of reflection spectra under linearly polarized forward and backward incidences are depicted in Figure S3. Meanwhile, the amplitude of the cross-polarized reflection coefficient under *y*-polarized incidence can reach more than 0.5 around the center frequency of 10 GHz as shown in Figures S3(a). The simulated phase different is exhibited in Figures S3(b), offering the basic effective microwave elements for full manipulation of the cross-polarized reflected wave. The slight shift existing in the phase response does not affect the performance of the metasurface.


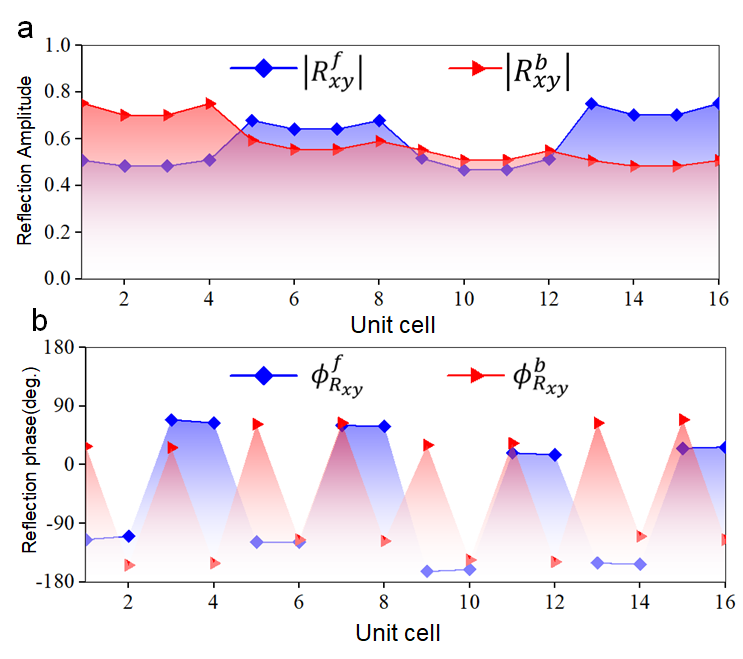


**Figure S3.** Cross-polarized reflection characteristics of all 16 coding atoms under y-polarized forward and backward incident waves. (a) and (b) Amplitude responses. (c) and (d) Phase responses.

In the main text, we have discussed the two application examples to verify the functionality of the metasurface. Here, we give further results as depicted in Figures S4 and S5 of the cross-polarized reflection energy distribution for the both forward and backward incident waves. To realize the desired focal patterns, the ideal continuous phase profiles are shown in Figures S4(a) and S4(d), and the simulation and experimental results are consistent as illustrated in Figures S4(b), S4(c), and S4(e), S4(f), indicating that the asymmetric focus pattern is realized by means of the directional characteristics of the element. Figures S5(a) and S5(d) show the ideal phase of the hologram images “O” and “E”, and the Figures S5(b), S5(e) and S5(c), S5(f) show the simulated and measured cross-polarized reflected wave when the metasurface is illuminated by *y*-polarized forward and backward incident waves. As shown by the results, the crosstalk between the channels of the holograms “O” and “E” is negligible.


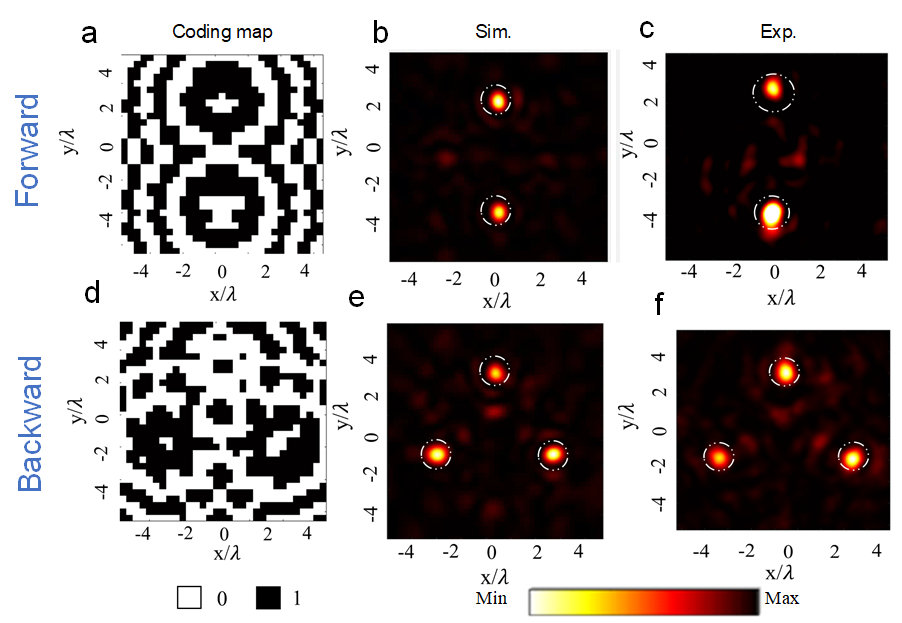


**Figure S4**. Cross-polarized reflection channel for linearly *y*-polarized forward and backward incident waves. (a) and (d) Phase distribution. (b) and (e) Simulated energy distribution. (c) and (f) Measured energy distribution.


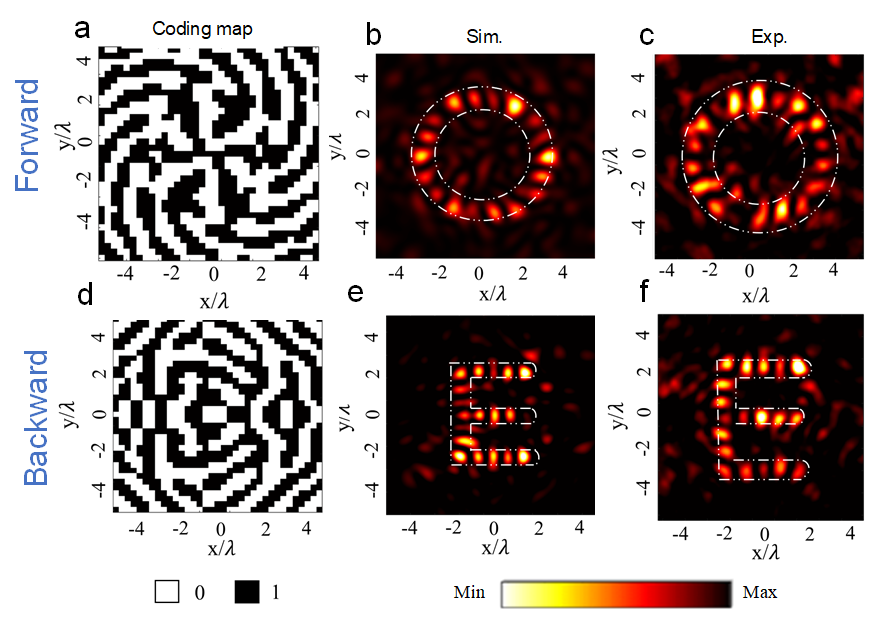


**Figure S5**. Cross-polarized reflection channel for linearly *y*-polarized forward and backward incident waves. (a) and (d) Phase distribution. (b) and (e) Simulated energy distribution. (c) and (f) Measured energy distribution.

**Text S4. Fabrication.**

The metasurface proposed in this article is manufactured by conventional printed circuit board (PCB) technology. For the multilayer metasurface, the metal surface treatment is first performed on the dielectric substrate with double-layer cladding to ensure that the surface of the substrate is free of oxide layer, oil, dust and other contaminants, so as to achieve the purpose of dry film attachment. Under the irradiation of ultraviolet light, the non-photosensitive dry film needs to be rinsed with a photoresist developer. After exposure, the metal under the photoresist is retained and is not affected by etching, and then the black oxide process for surface roughening and oxidation prevention is completed. For multi-layer samples, each treated copper-cladded dielectric board is glued together with an adhesive.

Based on such PCB technique, the metasurface samples are fabricated as shown in the Figure S6, where the insets show zoomed parts for a clear illustration. Blocked by the top layer, the grating structures in the intermediate layer cannot be shown. So we only show the top and bottom views of the metasurfaces.


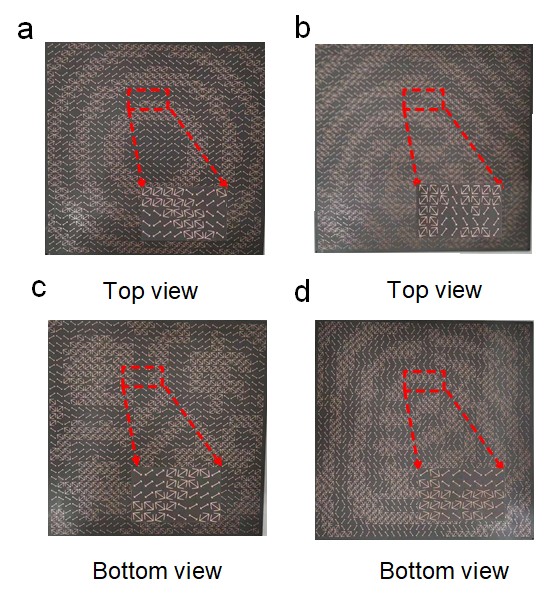


**Figure S6**. Top and bottom views of the fabricated samples showing multiple functions in co-polarized reflection and cross-polarized reflection and transmission. (a) and (c) Focusing. (b) and (d) Holograms of the letters of “LOVE”

For experimental demonstration, the field distribution in reflection and transmission wave have been experimentally measured by the near-field scanning system exhibited in Figure S7. Each port of the vector network analyzer is respectively connected to the illuminating horn antenna and detection probe. The horn antenna is placed far away from the metasurface sample to ensure a quasi-planar wavefront so as to mimic the plane wave incidence used in simulations. The two detection planes represent the reflection and transmission planes, which are placed at a distance of 80mm from the metasurface. The moving step of the translation stages on which is placed the probe is set as 4 mm to enable a sufficient resolution of the measured field distributions.


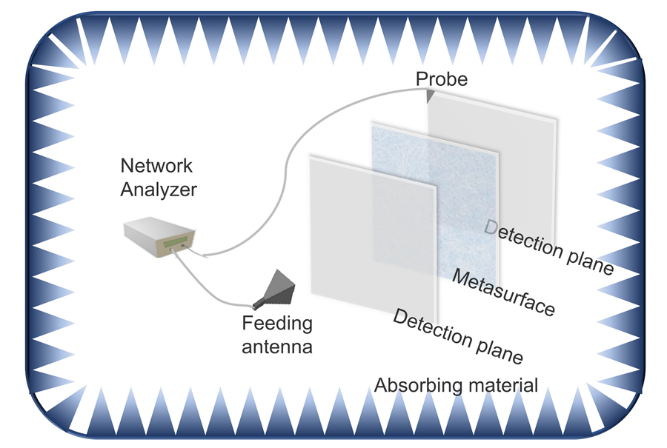


**Figure S7**. Near-field mapping measurement system.
